# The pilot study examining the effects of swallowing position on lung volume fraction and the coordination between respiration and non‐nutritive swallowing reflex

**DOI:** 10.1002/cre2.274

**Published:** 2020-02-17

**Authors:** Kaori Yamaguchi‐Komeyama, Terumi Ayuse, Shinya Mikushi, Noriko Hisamatsu, Taiki Yamaguchi, Nobuaki Magata, Naomi Tanoue, Hanako Kawasaki, Ryo Kozu, Hideaki Takahata, Takao Ayuse

**Affiliations:** ^1^ Department of Special Care Dentistry Nagasaki University Hospital of Medicine and Dentistry Nagasaki Japan; ^2^ Department of Dental Anesthesiology, Course of Medical and Dental Sciences Nagasaki University Institute of Biomedical Sciences Nagasaki Japan; ^3^ Department of Cardiopulmonary Rehabilitation Science Nagasaki University Graduate School of Biomedical Sciences Nagasaki Japan; ^4^ Department of Rehabilitation Medicine Nagasaki University Hospital Nagasaki Japan

**Keywords:** body position, respiratory function, swallowing apnea

## Abstract

**Background:**

Body position might affect the coordination between respiration and swallowing. This study was carried out to test the hypothesis that during swallowing, coordinated movements of muscle groups such as the diaphragm and rectus abdominis muscles are important to control normal swallowing apnea.

**Objective:**

To investigate this hypothesis, respiratory parameters, swallowing apnea and muscle activity were measured in each of four body positions: sitting position with feet on the floor, 30° reclining position, lateral position, and standing position.

**Methods:**

All measurements were performed in nine healthy subjects. Nasal airflow was measured using a pneumotachometer and muscle activity was measured using an electromyograph. All lung volume fraction parameters were measured using spirometer and swallowing apnea time was calculated.

**Results:**

The maximum inspiratory volume was 2.76 ± 0.83 L in the 30° reclining position, which was significantly larger than that in the other positions (*p* = .0001). The preliminary expiratory volume was 1.05 ± 0.42 L in the 30° reclining position, which was significantly smaller than that in the other positions (*p* < .0001). The swallowing apnea time during water swallowing was 1.17 ± 0.35 sec in the lateral position and 0.87 ± 0.28 sec in the 30° reclining position, which tended to be longer than the 0.78 sec in the sitting position.

**Conclusion:**

We conclude that both lateral and reclining positions require a longer period of swallowing apnea compared to the sitting and standing positions. Differences in body position may significantly influence the coordination between respiration and swallowing.

## 1 INTRODUCTION

The swallowing reflex is a complex reflex that involves coordinated contraction of several muscles in the oropharyngeal region. Although extensive experimental evidence supports the clinical observation of neuro‐physiological, structural, and functional interdependence between the upper airway system and swallowing function (Ayuse et al., 2006, 2010; Jafari, Prince, Kim, & Paydarfar, 2003; Kijima, Isono, & Nishino, 1999; Martin, Logemann, Shaker, & Dodds, 1994; Paydarfar, Eldridge, & Paydarfar, 1998; Preiksaitis, Mayrand, Robins, & Diamant, 1992; Preiksaitis & Mills, 1996). The highly coordinated interaction between respiration and swallowing is essential for the maintenance of adequate ventilation without causing pulmonary aspiration. The coordinated movement of the swallowing and breathing phase is physiologically adjusted so that swallowing does not occur during inspiration in the breathing cycle and secondly so that food or liquid does not accidentally flow into the airway during swallowing. The coordination between respiration and swallowing at meal is a very important factor for controlling physiological swallowing function, but this interaction might be easily influenced by the posture of the upper body, the head and neck. Ease of swallowing and ease of breathing are contradictory physiological effects. The head is raised to ensure sufficient breathing until just before swallowing, but in the swallowing process, the jaws are pulled, breathing is stopped (swallowing apnea).

Swallowing apnea is an important mechanism that prevents aspiration by physiologically separating inspiration and swallowing during the expiration phase or during the transition from the inspiration phase to the expiration phase. The breath‐holding function for swallowing depends on the respiratory cycle of the inspiratory phase and expiratory phase, so it may be greatly affected by respiratory parameters such as respiratory rate, tidal volume, lung volume, and forced expiratory volume in the first second. In addition, it is known that these parameters are greatly affected by body position, which is a problem in pathological conditions where the respiratory function is affected.

The pharyngeal muscles directly involved in swallowing play an important role in the regulatory mechanism of swallowing function, but the deep cervical flexor muscles (cervical neck) are involved in maintaining head and neck posture during swallowing. The trunk‐holding muscles and the respiratory assist muscles (thoracic chain mastoid) that regulate the respiratory cycle also have important functions, although indirectly. Among the muscle groups important for maintaining the posture of the trunk, the muscle groups that originate from the skull function mainly when performing anteroposterior flexion, lateral flexion, rotation of the head and neck and posture control of the head, neck and mandible. If these roles are abnormally inhibited, the coordination between swallowing function and respiration might be seriously affected. Therefore, if head and neck posture control and the maintenance function of the respiratory cycle are not adjusted at an extremely precise timing, smooth swallowing cannot be performed. It has been generally recognized that a functional decline of the deep flexor muscles in the head and neck greatly affects the activity of the sternocleidomastoid muscle, a respiratory assist muscle, and that the interaction between both muscles is particularly important. However, the interaction between the two muscle groups is not clear, and no studies have examined its function.

During swallowing, in addition to the muscle groups directly involved such as the suprahyoid muscle group, the head and neck deep flexor muscle group (cervical and longitudinal muscles) that regulates head and neck flexion, and the respiratory assist muscle that regulates the respiratory phase is also important. During swallowing, the control function of swallowing apnea is important to prevent aspiration, and the function of muscle groups related to breath holding in the expiratory phase is easily affected by lung volume fractionation caused by changes in body position. The interaction between the two muscle groups is particularly important, as it has been shown that the functional decline of the deep flexor muscles of the head and neck greatly affects the activity of the sternocleidomastoid muscle, which is a respiratory assist muscle (Matsuo, Hiiemae, Gonzalez‐Fernandez, & Palmer, 2008).

In this study, we investigated the effects of swallowing position on breath‐holding lung volume, muscle activity in breath‐related muscle groups, and swallowing apnea time in the respiratory cycle. We hypothesized that during swallowing, coordinated movements of muscle groups such as the diaphragm and rectus abdominis muscles are important to control normal swallowing apnea. If there is a change in lung volume, the resulting swallowing/respiratory coordination is affected.

## 2 SUBJECTS AND METHODS

### 2.1 Subjects criteria

All measurements were performed in nine healthy subjects (four males, five females), who were 22.2 ± 3.0 years of age and had a mean body weight of 56.0 ± 9.6 kg, height of 1.60 ± 0.1 m and body mass index (BMI) of 20.9 ± 2.6 kg/m^2^. Subjects who reported a history of dysphagia or anatomical deformation in the oropharyngeal region were excluded from this study. The protocol was approved by the Human Investigation Committee of the Nagasaki University and written informed consent was obtained from all subjects.

### 2.2 Measurement of respiratory parameters

Nasal airflow was measured using a pneumotachometer (model 3830, Hans Rudolph, Inc., Kansas City, MO) and nasal pressure (*P*_N_) was measured using a differential pressure transducer (model 1100, Hans Rudolph, Inc., Kansas City, MO).

### 2.3 Swallowing apnea

Duration of swallowing apnea was assessed by measuring the plateau phase (inspiratory zero airflow) on the respiratory trace (nasal inflow) as described in previous study (Ayuse et al., 2010).

### 2.4 Timing of swallow in relation to respiratory cycle phase

Timing of swallows in relation to respiratory cycle phase was evaluated in each condition. Swallows preceded by and followed by inspiratory flow were marked as inspiratory swallows (I‐I), whereas swallows preceded by and followed by expiration flow were designated expiratory swallows (E‐E). Swallows occurring at the transition between inspiration and expiration were designated inspiratory–expiratory (I‐E) transition swallows, and those occurring at the transition between expiration and the inspiratory phase of the next breath were designated expiratory–inspiratory (E‐I) transition swallows.

### 2.5 Measurement of muscular activity

**Figure 1:**
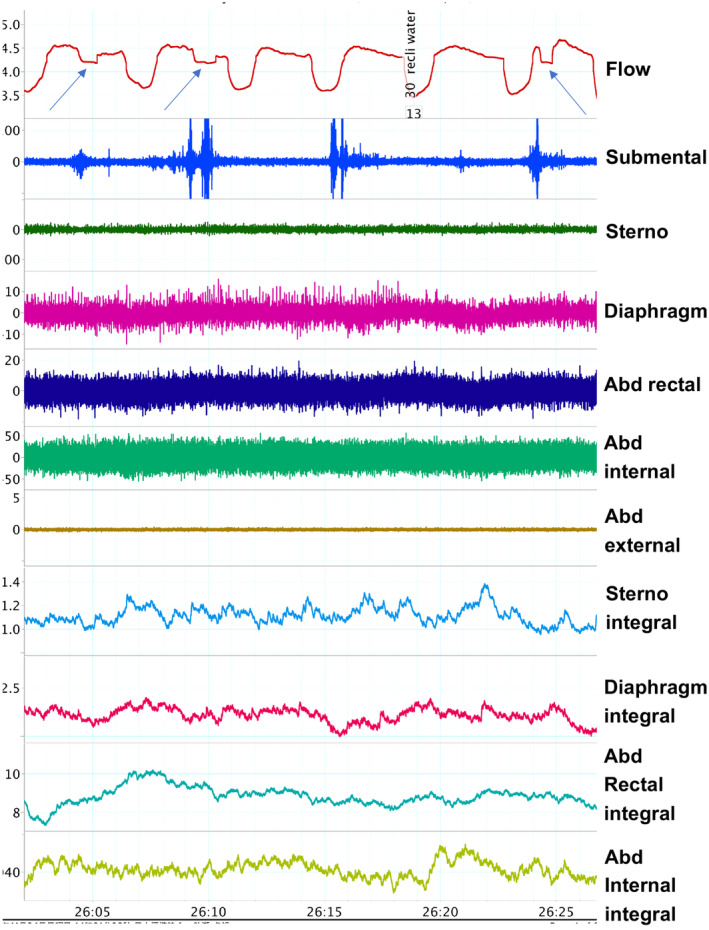
Representative trace of all parameters (flow, raw EMGs and integrated EMGs) in a 30° reclining position during water swallowing. The arrow indicate the occurrence of swallowing apnea

Surface electromyograms (EMGs) were recorded from the submental muscles (Bagnoli EMG system, manufactured by DELSYS). Electrodes were placed 1 cm posterior to the genu of the mandible over the midline suprahyoid muscle complex with an interelectrode distance of 3 cm. We also recorded EMGs from diaphragm, sternocleidomastoid muscles as inspiratory control muscles, a rectus abdominal muscles as expiratory control muscles, a medical oblique muscles and lateral oblique muscles (Figure 1). These signals were differentially amplified (Bioamp PowerLab) relative to a surface recording over the left zygomatic arch. An electrode over the right zygomatic arch served as the ground lead. The EMG signal was full wave rectified, and integrated in 50 ms intervals. The measurement was performed after confirming that the impedance test built in the electromyograph was cleared. The data measured from the electromyograph was A/D converted at a sampling frequency of 1 kHz and loaded into an analysis computer. The waveform analysis was carried out using myoelectric analysis software. The bandpass filter was set to between 10 and 500 Hz. At the peak of muscle activity, rest was taken as the zero reference.

### 2.6 Measurement of lung volume fraction

**Figure 2:**
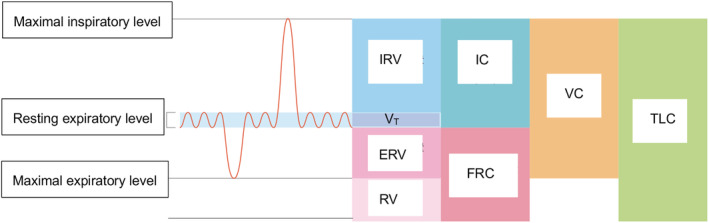
Diagram of spirogram indicating the parameters of lung volume fraction*,* vital capacity (VC), tidal volume (*V*_T_), reserve inspiratory volume (IRV), reserve expiratory volume (ERV), and maximum inspiratory volume (IC)

We calculated vital capacity (VC), tidal volume (*V*_T_), reserve inspiratory volume (IRV), reserve expiratory volume (ERV), and maximum inspiratory volume (IC), which is an index of shortness of breath and exercise tolerance using spirogram (Figure 2). Measurements for each subject were carried out during separate experiments and using a spirometer prior to the swallowing protocol.

### 2.7 Experimental protocol and body position

Body postures included four positions: sitting position with feet on the floor, 30° reclining from floor position, lateral position and standing position. Swallowing was performed with drinking of water (20 ml) and jelly intake. The primary endpoint was the reserve expiratory volume (ERV), maximum inspiratory volume (IC), swallowing apnea time, and rate change in muscle activity in the breath‐related group at each position.

### 2.8 Data analysis

Diaphragm muscle activity represents %muscle activity based on the 3‐sec integrated value of electrocardiogram during inspiration, and the rectus abdominis and internal oblique muscles are raised and laterally exercised. Relative muscle activity at each time point of food intake for each body posture was calculated based on the muscle activity associated with 100% muscle activity.

### 2.9 Statistical analysis

Results are expressed with mean ± *SD*. For statistical analysis, a one‐way repeated measure of ANOVA test was performed with turkey post hoc test. *p* < .05 was set as statistically significant.

## 3 RESULTS

### 3.1 Swallowing apnea

**Figure 3:**
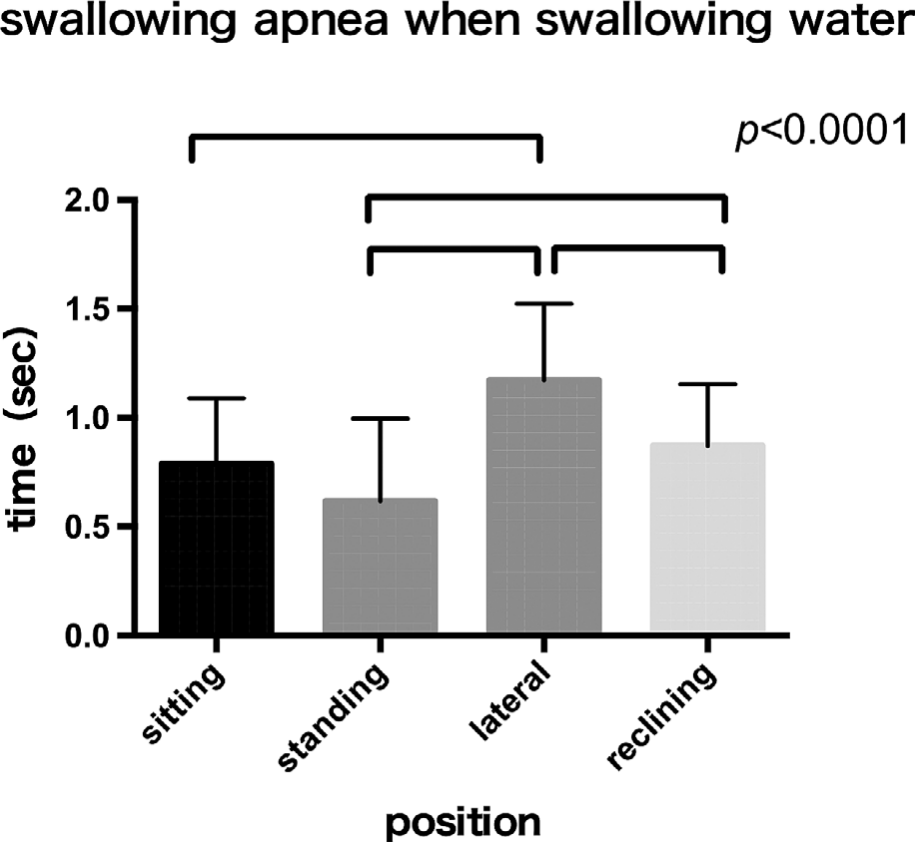
There is a significant increase in swallowing apnea at water swallow in the lateral position compared to the sitting, standing and reclining position. (*p* < .0001)

**Figure 4:**
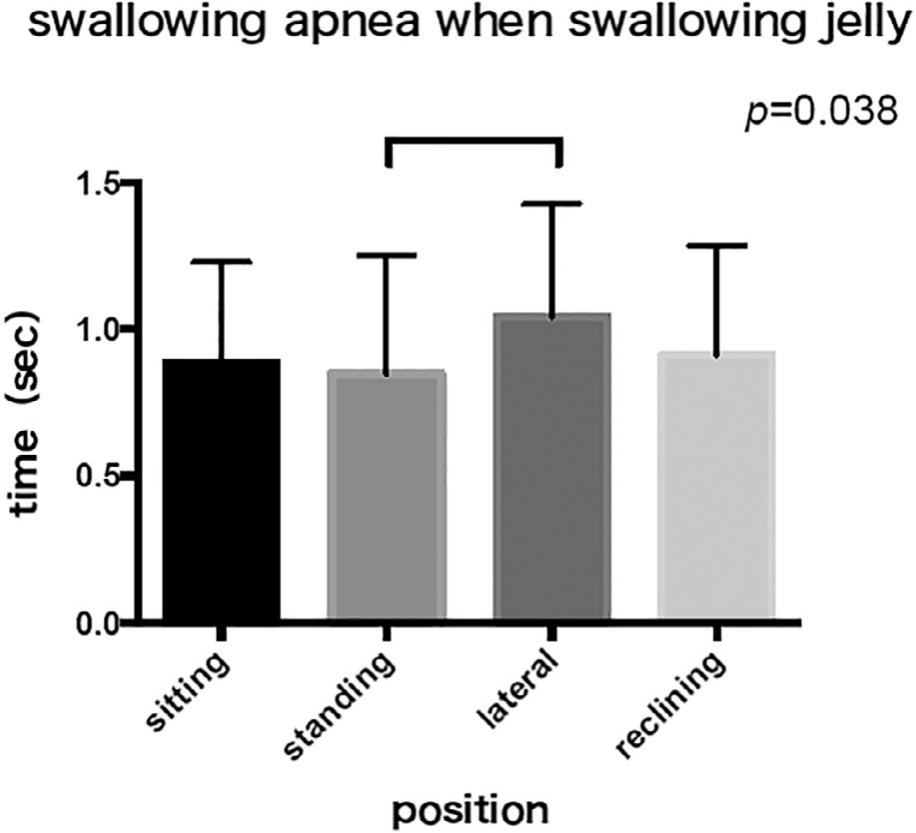
There is a significant increase in swallowing apnea at jelly swallow in the lateral position compared to the sitting, standing and reclining position. (*p* = .038)

The mean swallowing apnea time during water swallowing was 1.17 ± 0.35 sec in the lateral position and 0.87 ± 0.28 sec in the 30° reclining position, longer than the 0.78 ± 0.30 sec in the sitting position (Figure 3). Divided swallowing was frequently observed in the lateral position. The mean swallowing apnea time during jelly swallowing was 1.05 ± 0.39 sec in the lateral position, which tended to be longer than the 0.88 ± 0.34 sec in the sitting position and the 0.88 ± 0.38 sec in the 30° reclining position (Figure 4).

### 3.2 Respiratory parameters

**Figure 5:**
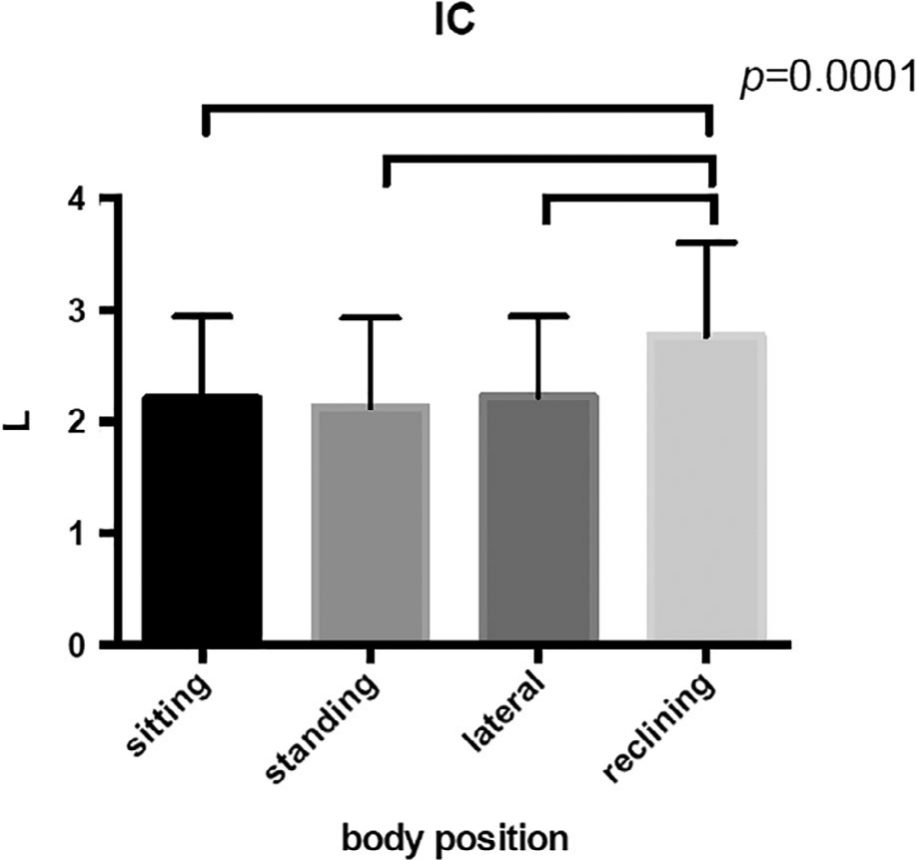
There is a significant increase in maximum inspiratory volume (IC) in the reclining position compared to the sitting, standing and lateral position. (*p* < .0001)

**Figure 6:**
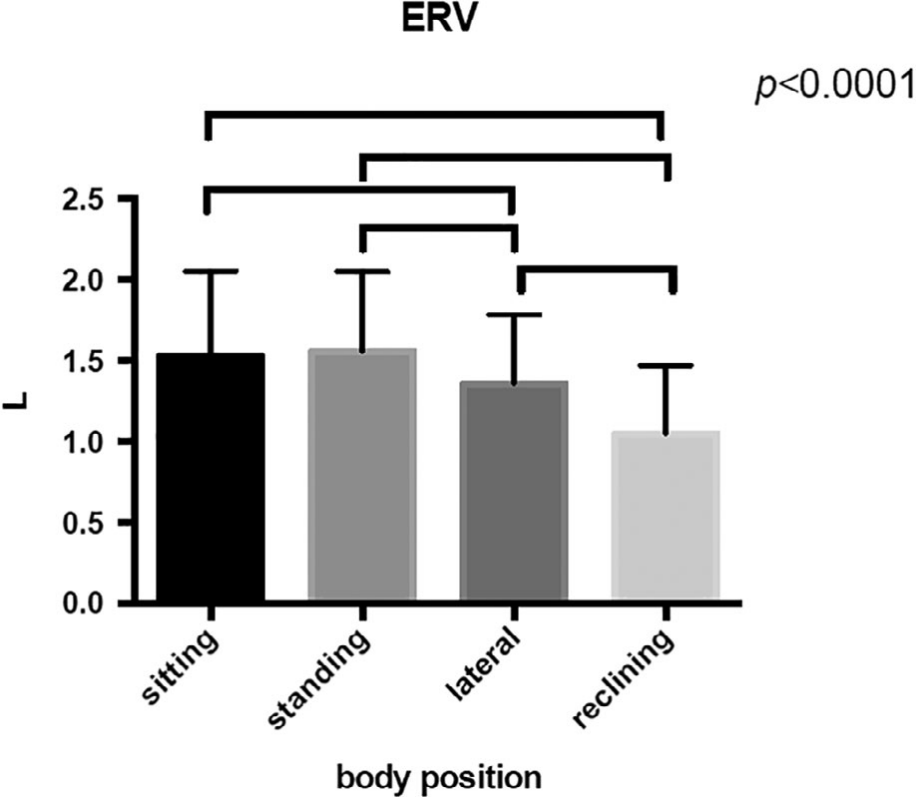
There is a significant increase in reserve expiratory volume (ERV) in the reclining position compared to the sitting, standing and lateral position. (p < 0.0001)

All the parameters of lung volume fraction was obtained at different posture in each subjects (Table 1). The maximum inspiratory volume (IC) was at 30° reclining position (2.76 ± 0.83 L), significantly higher than in other positions (*p* = .0001; Figure 5). The smallest preliminary expiratory volume (ERV) was at 30° reclining position (1.05 ± 0.42 L), significantly smaller than in other positions (*p* < .0001; Figure 6). Otherwise, there was no significant difference in respiratory rate (*f*), vital capacity (VC), tidal volume (*V*_T_) and reserve inspiratory volume (IRV) among the positions.

**Table 1:** There is a significant reduction in ERV in the lateral and reclining position

Parameters	Sitting	Standing	Lateral	Reclining	*p* value
*f*	12.3 ± 3.34	14.0 ± 2.95	13.8 ± 3.10	14.1 ± 2.26	.420
VC	3.75 ± 0.95	3.73 ± 0.88	3.60 ± 1.00	3.65 ± 1.00	.079
*V*_T_	0.68 ± 0.21	0.73 ± 0.23	0.63 ± 0.16	0.70 ± 0.25	.770
IC	2.21 ± 0.74	2.13 ± 0.80	2.22 ± 0.72	2.76 ± 0.83	.0001
IRV	1.54 ± 0.61	1.45 ± 0.65	1.66 ± 0.65	1.83 ± 0.59	.492
ERV	1.53 ± 0.53	1.55 ± 0.49	1.34 ± 0.43	1.05 ± 0.42	*<.0001*

*Note: p* < .0001 reclining position versus sitting and standing, lateral position versus sitting and standing, reclining position versus lateral.

### 3.3 Timing of swallow in relation to respiratory cycle phase

**Figure 7:**
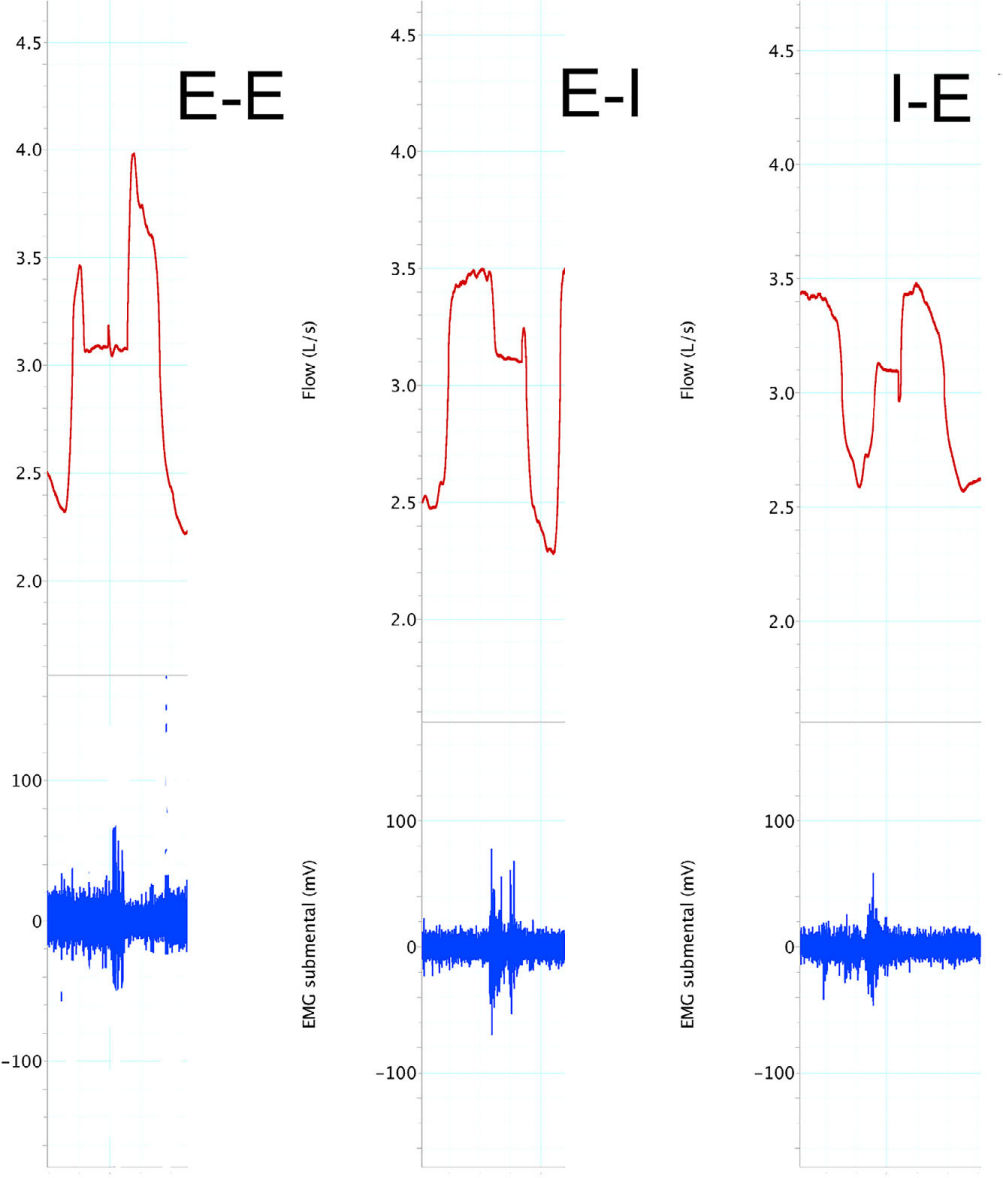
Representative trace of timing of swallowing at water swallow in one subject

There was no significant difference of timing of swallow among position both in jelly swallowing and water swallowing (Table 2). There was different timing of swallowing due to different posture and different bolus type in each subjects (Figure 7).

**Table 2:** Timing of swallowing in water and jelly swallow

Jelly				
	Sitting	Standing	Lateral	Reclining
E‐E type (%)	78.2 ± 9.2	62.2 ± 22.1	71.9 ± 10.9	69.7 ± 11.9
E‐I type (%)	17.0 ± 7.7	16.1 ± 23.5	18.4 ± 10.8	15.3 ± 12.3
I‐E type (%)	7.6 ± 9,6	20.3 ± 18.3	8.3 ± 10.8	15.3 ± 11.9
I‐I type (%)	0.0	0.0	0.0	0.0

Water				
	Sitting	Standing	Lateral	Reclining
E‐E type (%)	83.4 ± 5.8	67.5 ± 23.1	75.8 ± 11.4	71.8 ± 9.2
E‐I type (%)	10.5 ± 5.8	20.5 ± 25.0	21.0 ± 10.8	23.6 ± 12.8
I‐E type (%)	5.8 ± 5.4	14.4 ± 16.0	4.6 ± 6.7	6.9 ± 6.7
I‐I type (%)	0.0	0.0	0.0	0.0

*Note:* There was no significant difference in swallowing jelly and water timing among position.

### 3.4 EMGs activity

**Figure 8:**
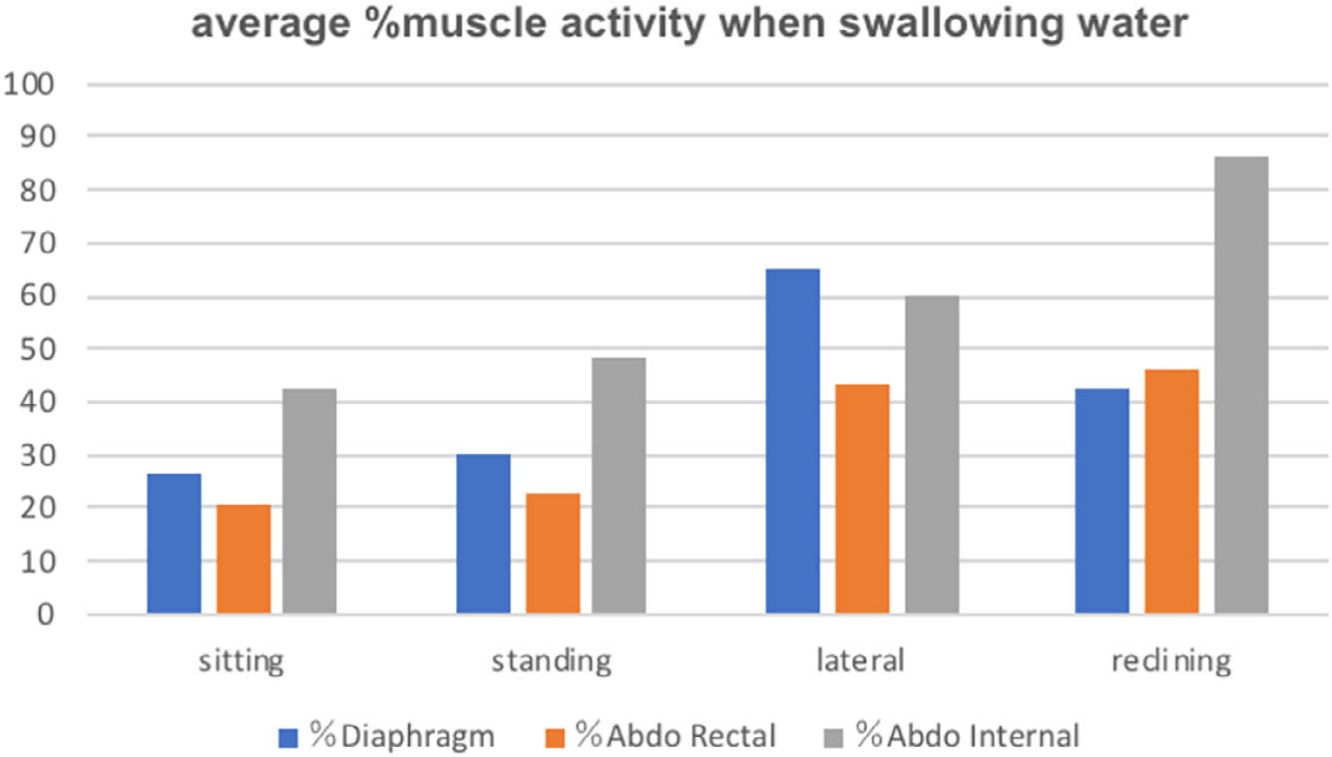
There is a tendency of increase in %diaphragm activity in a lateral position and a tendency of increase in %abdo internal reclining position at water swallow

**Figure 9:**
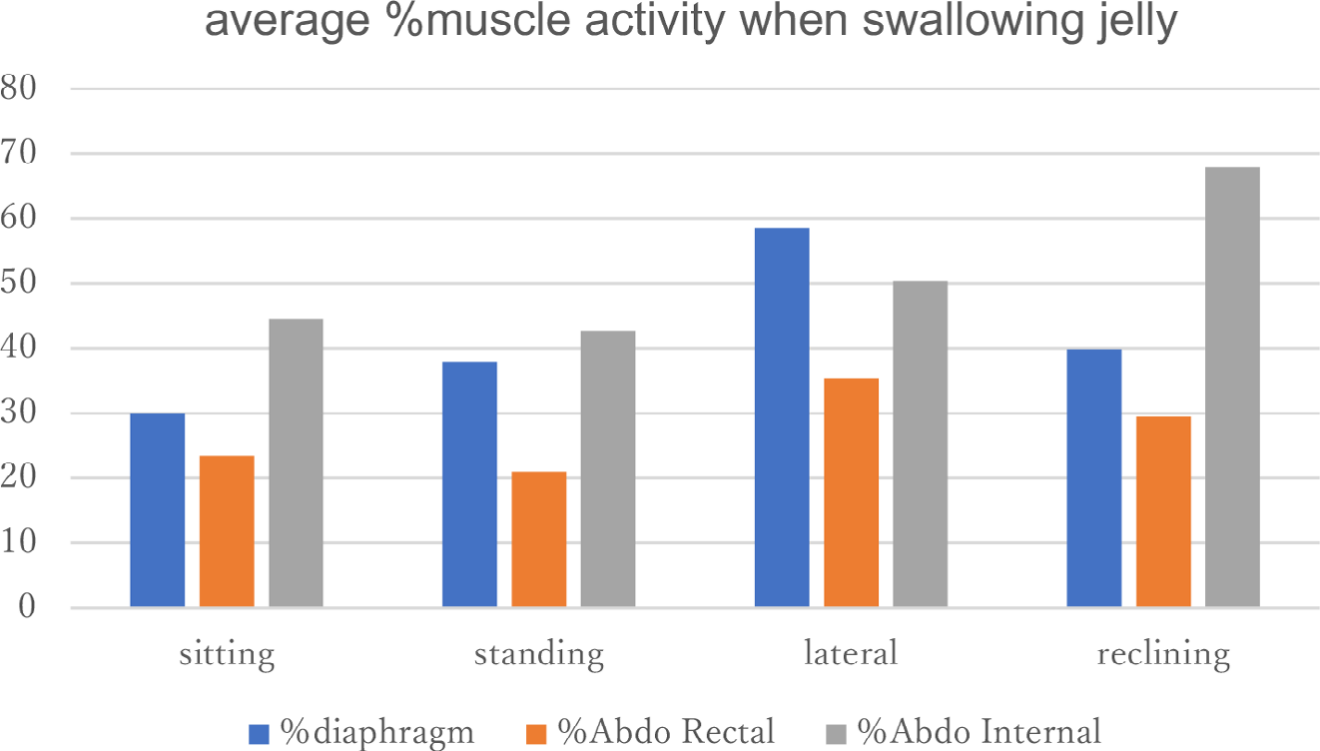
There is a tendency of increase in %diaphragm EMGs activity in a lateral position and a tendency of increase in %abdo EMGs activity internal reclining position at jelly swallow

The muscle activity of the diaphragm and the muscle activity of the rectus abdominal muscle and the internal oblique muscle tends to be higher in the lateral position and in the 30° reclining position compared to the standing and sitting positions (Figures 8 and 9).

## 4 DISCUSSION

The major findings of this study are (a) in the lateral recumbent position the muscle activity of the diaphragm is greater compared to the standing and sitting positions, (b) in the 30° reclining position the muscle activity of the Abdominal internal is greater compared to the standing and sitting positions, (b) changes in respiratory function, such as maximum inspiratory volume (IC), due to postural changes may affect the modulation of breath‐holding function during swallowing, resulting in the possibility of prolonging swallowing apnea time.

IC (maximum inspiratory volume) is one of the indicators of lung volume fraction, together with *V*_T_ (tidal volume), IRV (inspiratory reserve volume), FRC (functional residual capacity), and TLC (total lung volume). Corresponding to the lung volume fraction (preliminary inspiratory volume), COPD patients are more strongly associated with clinical indicators such as shortness of breath than FEV1%. During swallowing, coordination of muscle groups such as the diaphragm and rectus abdominis muscles associated with breath holding is important to control normal swallowing apnea. However, if there is dysfunction of the breath holding muscle group, swallowing and respiratory coordination is impaired due to changes in lung volume (Kijima et al., 1999; Kijima, Isono, & Nishino, 2000).

### 4.1 Swallowing apnea

Recently, Olsson, Castell, Johnston, Ekberg, and Castell (1997) described laryngeal elevation as the single most important factor for a successful pharyngeal swallowing sequence. The major purpose of laryngeal elevation for respiratory function is thought to be opening the upper esophageal sphincter associated with airway closure during swallowing, that is, deglutition apnea (Matsuo et al., 2008). Duration of swallowing apnea has been measured in several studies and mean duration appears to be between 0.6 and 0.76 sec in young subjects (Klahn & Perlman, 1999; Perlman, Ettema, & Barkmeier, 2000; Selley, Flack, Ellis, & Brooks, 1989) and 0.6 sec in older subjects (Hirst, Ford, Gibson, & Wilson, 2002). In this study, duration of swallowing apnea obtained in the sitting position during water intake was 0.78 ± 0.30 sec and is therefore consistent with previous data. In our study, there was an increase in the duration of swallowing apnea from 0.78 sec with sitting position to 1.17 sec with lateral position and to 0.87 sec with 30° reclining position. A similar trend of increasing apnea duration was also found by Perlman et al. (2000) in younger subjects with increasing bolus volume. Possible mechanisms causing prolongation of apnea with increasing bolus volume have been previously discussed by Ertekin et al. (1997), who concluded that this phenomenon is related to increased duration of submental EMG activity, UES opening and laryngeal displacement. Recently Hiss, Strauss, Treole, Stuart, and Boutilier (2003) suggested that swallowing apnea may occur secondary to a specific neural command from the brainstem but may also be a habituated motor response. We speculate that increased diaphragm EMG activity with alteration of lung volume fraction due to posture change might affect neural command or motor response.

### 4.2 EMG activity in swallowing muscles

In this study, we observed an increase in EMG activity of the diaphragm in the lateral position associated with a prolongation of swallowing apnea time. However, we did not find any significant change in the activity of other muscles, even though there was a tendency of the activity to increase in the lateral and reclining position compared to the sitting position. We hypothesize that this alteration is important to avoid aspiration of water or jelly, based on the normal range of respiratory rate in healthy subjects. However, if respiratory rate had already been increased due to changes in respiratory function like in obstructive respiratory diseases such as COPD, the compensatory controls of alteration of swallowing apnea and increased EMG activity would not be functional.

### 4.3 Methodological limitations

The present study has several methodological limitations. First, we performed these evaluations in a limited number of normal healthy volunteers in three different body position conditions. It is clear that simple extrapolation of our results to patients with respiratory disease may not be entirely valid. For instance, the effect of posture on the coordination between respiration and non‐nutritive swallowing may differ from that observed in elderly subjects or patients.

Second, we should consider the timing of swallowing. In line with previous studies (Nishino, Yonezawa, & Honda, 1985; Preiksaitis et al., 1992; Shaker et al., 1992; Smith, Wolkove, Colacone, & Kreisman, 1989), we observed that, in the lateral position, the majority of swallows interrupted breathing in the expiratory phase (E–E) because of prolongation of swallowing apnea. In this study, we did not observe any significant difference of the timing of swallowing in each posture condition, even though we observed the occurrence of swallowing in the I‐E phase and split swallowing in lateral position. The occurrence of this interruption in the I‐E transition increased in the lateral position. We speculate that prolongation of swallowing apnea and increased EMGs activity might compensate the alteration of lung volume fraction caused by posture change. Although we do not know whether this evaluation is a useful method for assessing the timing of swallowing in the lateral and reclining position, these results may have important clinical implications.

### 4.4 Clinical implications and future research

We may conclude that alterations in respiratory function due to changes in lung volume fraction and respiratory rate depend on body position and might affect the coordination between swallowing and respiratory movements. Since this study was performed in normal young healthy subjects, we cannot infer whether this lack of coordination is critical in dysphagia patients. However, we can speculate that changes affecting the coordination between respiration and swallowing might be potentiated in patients with upper airway dysfunction, such as COPD patients who have an increased respiratory rate, (Cvejic et al., 2011; Gross, Atwood Jr., Ross, Olszewski, & Eichhorn, 2009) or respiratory disease (Kijima et al., 1999, 2000). During swallowing, in addition to the muscle groups directly involved such as the suprahyoid muscle group and the head and neck deep flexor muscle group (cervical and longitudinal muscles) that regulate head and neck flexion, the respiratory assist muscle that regulates the respiratory phase (involvement of the sternocleidomastoid muscle, oblique muscle) is also important. During swallowing, it is important to regulate swallowing apnea to prevent aspiration, and the functions of muscle groups related to breath holding in the expiratory phase are likely to be affected by lung volume fractions due to postural changes (Alghadir, Zafar, Al‐Eisa, & Iqbal, 2017; Calvo, Sunday, Macrae, & Humbert, 2017). Although a simple extrapolation of the results of our study to other clinical situations may not be entirely valid, future work is required to investigate the pathogenesis of dysphagia due to malfunction of the coordination between respiration and swallowing in respiratory disease.

In conclusion, in the lateral recumbent position and in the 30° reclining position the muscle activity of the diaphragm and the muscle activity of the rectus abdominal muscle and the internal oblique muscle are greater compared to in other standing and sitting positions. Changes in respiratory function, such as maximum inspiratory volume (IC), due to postural changes may affect the modulation of breath‐holding function during swallowing, resulting in the possibility of prolonged swallowing apnea time.

## References

[cre2274-bib-0001] Alghadir, A. H. , Zafar, H. , Al‐Eisa, E. S. , & Iqbal, Z. A. (2017). Effect of posture on swallowing. African Health Sciences, 17(1), 133–137. 10.4314/ahs.v17i1.17 29026386 PMC5636236

[cre2274-bib-0002] Ayuse, T. , Ayuse, T. , Ishitobi, S. , Kurata, S. , Sakamoto, E. , Okayasu, I. , & Oi, K. (2006). Effect of reclining and chin‐tuck position on the coordination between respiration and swallowing. Journal of Oral Rehabilitation, 33(6), 402–408. 16671985 10.1111/j.1365-2842.2005.01586.x

[cre2274-bib-0003] Ayuse, T. , Ayuse, T. , Ishitobi, S. , Yoshida, H. , Nogami, T. , Kurata, S. , … Oi, K. (2010). The mandible advancement may alter the coordination between breathing and the non‐nutritive swallowing reflex. Journal of Oral Rehabilitation, 37(5), 336–345. 10.1111/j.1365-2842.2010.02067.x 20337868

[cre2274-bib-0004] Calvo, I. , Sunday, K. L. , Macrae, P. , & Humbert, I. A. (2017). Effects of chin‐up posture on the sequence of swallowing events. Head & Neck, 39(5), 947–959. 10.1002/hed.24713 28181331 PMC5435481

[cre2274-bib-0005] Cvejic, L. , Harding, R. , Churchward, T. , Turton, A. , Finlay, P. , Massey, D. , … Guy, P. (2011). Laryngeal penetration and aspiration in individuals with stable COPD. Respirology, 16(2), 269–275. 10.1111/j.1440-1843.2010.01875.x 21054669

[cre2274-bib-0006] Ertekin, C. , Aydogdu, I. , Yuceyar, N. , Pehlivan, M. , Ertas, M. , Uludag, B. , & Celebi, G. (1997). Effects of bolus volume on oropharyngeal swallowing: An electrophysiologic study in man. The American Journal of Gastroenterology, 92(11), 2049–2053. 9362190

[cre2274-bib-0007] Gross, R. D. , Atwood, C. W. Jr. , Ross, S. B. , Olszewski, J. W. , & Eichhorn, K. A. (2009). The coordination of breathing and swallowing in chronic obstructive pulmonary disease. American Journal of Respiratory and Critical Care Medicine, 179(7), 559–565. 10.1164/rccm.200807-1139OC 19151193

[cre2274-bib-0008] Hirst, L. J. , Ford, G. A. , Gibson, G. J. , & Wilson, J. A. (2002). Swallow‐induced alterations in breathing in normal older people. Dysphagia, 17(2), 152–161. 11956841 10.1007/s00455-001-0115-3

[cre2274-bib-0009] Hiss, S. G. , Strauss, M. , Treole, K. , Stuart, A. , & Boutilier, S. (2003). Swallowing apnea as a function of airway closure. Dysphagia, 18(4), 293–300. 14571335 10.1007/s00455-003-0021-y

[cre2274-bib-0010] Jafari, S. , Prince, R. A. , Kim, D. Y. , & Paydarfar, D. (2003). Sensory regulation of swallowing and airway protection: A role for the internal superior laryngeal nerve in humans. The Journal of Physiology, 550(Pt. 1), 287–304. 12754311 10.1113/jphysiol.2003.039966 PMC2343009

[cre2274-bib-0011] Kijima, M. , Isono, S. , & Nishino, T. (1999). Coordination of swallowing and phases of respiration during added respiratory loads in awake subjects. American Journal of Respiratory and Critical Care Medicine, 159(6), 1898–1902. 10351937 10.1164/ajrccm.159.6.9811092

[cre2274-bib-0012] Kijima, M. , Isono, S. , & Nishino, T. (2000). Modulation of swallowing reflex by lung volume changes. American Journal of Respiratory and Critical Care Medicine, 162(5), 1855–1858. 11069826 10.1164/ajrccm.162.5.2005106

[cre2274-bib-0013] Klahn, M. S. , & Perlman, A. L. (1999). Temporal and durational patterns associating respiration and swallowing. Dysphagia, 14(3), 131–138. 10341108 10.1007/PL00009594

[cre2274-bib-0014] Martin, B. J. , Logemann, J. A. , Shaker, R. , & Dodds, W. J. (1994). Coordination between respiration and swallowing: Respiratory phase relationships and temporal integration. Journal of Applied Physiology, 76(2), 714–723. 8175582 10.1152/jappl.1994.76.2.714

[cre2274-bib-0015] Matsuo, K. , Hiiemae, K. M. , Gonzalez‐Fernandez, M. , & Palmer, J. B. (2008). Respiration during feeding on solid food: Alterations in breathing during mastication, pharyngeal bolus aggregation, and swallowing. Journal of Applied Physiology (Bethesda, MD: 1985), 104(3), 674–681. 10.1152/japplphysiol.00527.2007 18162483

[cre2274-bib-0016] Nishino, T. , Yonezawa, T. , & Honda, Y. (1985). Effects of swallowing on the pattern of continuous respiration in human adults. The American Review of Respiratory Disease, 132(6), 1219–1222. 2934012 10.1164/arrd.1985.132.6.1219

[cre2274-bib-0017] Olsson, R. , Castell, J. , Johnston, B. , Ekberg, O. , & Castell, D. O. (1997). Combined videomanometric identification of abnormalities related to pharyngeal retention. Academic Radiology, 4(5), 349–354. 9156231 10.1016/s1076-6332(97)80116-x

[cre2274-bib-0018] Paydarfar, D. , Eldridge, F. L. , & Paydarfar, J. A. (1998). Phase resetting of the respiratory oscillator by carotid sinus nerve stimulation in cats. The Journal of Physiology, 506(Pt. 2), 515–528. 9490875 10.1111/j.1469-7793.1998.515bw.x PMC2230724

[cre2274-bib-0019] Perlman, A. L. , Ettema, S. L. , & Barkmeier, J. (2000). Respiratory and acoustic signals associated with bolus passage during swallowing. Dysphagia, 15(2), 89–94. 10758191 10.1007/s004550010006

[cre2274-bib-0020] Preiksaitis, H. G. , Mayrand, S. , Robins, K. , & Diamant, N. E. (1992). Coordination of respiration and swallowing: Effect of bolus volume in normal adults. The American Journal of Physiology, 263(3 Pt. 2), R624–R630. 1415651 10.1152/ajpregu.1992.263.3.R624

[cre2274-bib-0021] Preiksaitis, H. G. , & Mills, C. A. (1996). Coordination of breathing and swallowing: Effects of bolus consistency and presentation in normal adults. Journal of Applied Physiology, 81(4), 1707–1714. 8904590 10.1152/jappl.1996.81.4.1707

[cre2274-bib-0022] Selley, W. G. , Flack, F. C. , Ellis, R. E. , & Brooks, W. A. (1989). Respiratory patterns associated with swallowing: Part 2. Neurologically impaired dysphagic patients. Age and Ageing, 18(3), 173–176. 2782214 10.1093/ageing/18.3.173

[cre2274-bib-0023] Shaker, R. , Li, Q. , Ren, J. , Townsend, W. F. , Dodds, W. J. , Martin, B. J. , … Rynders, A. (1992). Coordination of deglutition and phases of respiration: Effect of aging, tachypnea, bolus volume, and chronic obstructive pulmonary disease. The American Journal of Physiology, 263(5 Pt. 1), G750–G755. 1443150 10.1152/ajpgi.1992.263.5.G750

[cre2274-bib-0024] Smith, J. , Wolkove, N. , Colacone, A. , & Kreisman, H. (1989). Coordination of eating, drinking and breathing in adults. Chest, 96(3), 578–582. 2766816 10.1378/chest.96.3.578

